# Systemic Expression Analysis Reveals Prognostic Significance of WIPI3 in Hepatocellular Carcinoma

**DOI:** 10.3389/fgene.2020.00847

**Published:** 2020-08-20

**Authors:** Tao-tao Liang, Qi Shao, Zhi-chao Deng, Ting Wang, Qiao-zhen Kang

**Affiliations:** School of Life Sciences, Zhengzhou University, Zhengzhou, China

**Keywords:** hepatocellular carcinoma, WIPI3, prognosis, mutation, EIF4A3

## Abstract

**Introduction:**

WD repeat domain phosphoinositide-interacting protein 3 (WIPI3) is a member of the WIPI protein family, autophagy marker, that is associated with the malignant progression of various human cancers, but its role in hepatocellular carcinoma (HCC) is still unclear.

**Materials and Methods:**

Firstly, we collected the mRNA expression of WIPI3 in HCC through the platform of Oncomine, as well as the DNA copy number variations (CNVs), and verified it on human HCC cell line and the GEO database. Then, the subgroups and prognosis of HCC were performed by the UALCAN web tool. The mutation of WIPI3 was analyzed by cBioPortal. The coexpression of WIPI3 in HCC was identified from the LinkedOmics database, and function enrichment analysis was done using the LinkFinder module in LinkedOmics. Coexpression gene network was constructed through the STRING database, and the MCODE plug-in of which was used to build the gene modules; both of them were visualized by the Cytoscape software. Finally, the top modular genes in the same patient cohort were constructed through data mining in The Cancer Genome Atlas (TCGA) liver hepatocellular carcinoma (LIHC) by using the UCSC Xena browser.

**Results:**

The results indicated that WIPI3 was frequently overexpressed in HCC, which could lead to a poor prognosis through the Kaplan–Meier (KM) analysis. Moreover, there existed mutations of WIPI3 in HCC, and the prognosis of WIPI3-altered group was significantly poor based on KM plotter data. Coexpression analysis showed that the coexpression gene of WIPI3 was associated with cell cycle and spliceosome. Further analysis suggested that WIPI3 and eukaryotic translation initiation factor 4A3 (EIF4A3) coordinately regulated the cancer cell cycle by spliceosome as a result of the strong positive correlation between them.

**Conclusion:**

In summary, WIPI3 is constantly overexpressed in HCC tissues, resulting in a poor prognosis; therefore, we can identify it as an effective target for the treatment of HCC.

## Introduction

Hepatocellular carcinoma (HCC), with a high recurrence rate of 70%, is a leading cause of cancer-related mortality worldwide, as well as the most frequent liver cancer (Ferlay et al., 2015). Due to the high proportion (90%) of HCC in all primary liver malignancies, it causes a severe threat to health and safety of humans all over the world (Galle et al., 2018). In genetic background, the occurrence of HCC is determined by the interaction among multiple genetic factors (Sanyal et al., 2010). Therefore, its pathogenesis controlled by the function of different genes and the interaction in different processes, such as signal transduction and cell cycle, is very complex. Previous studies have found that genetic molecular aberrations may cause HCC onset, including oxidative stress, cellular senescence, inflammatory response, and so on (Ramakrishna et al., 2013). However, different studies usually yield various results, and the underlying mechanism of HCC initiation and development remains unclear. Nowadays, the treatment of HCC includes chemotherapy, surgical resection, and liver transplantation; existing targeted drugs show unsatisfactory efficacy (Takiya et al., 1996; Boucher et al., 2002; Shen et al., 2016). No specific treatment strategy has been developed that focuses on HCC. Therefore, there is an urgent need to find the reliable biomarkers for diagnosis at early stage, judgment in prognosis, and effective treatment of HCC.

Autophagy is a highly conservative self-digestion process in eukaryotes and is crucial for cellular homeostasis, protein quality control, and pathogen defense (Mizushima et al., 2011; Wen and Klionsky, 2016), which often occurs during tumorigenesis and cancer chemotherapy. Generally, constructive autophagy protects cancer cells during chemotherapy, leading to cancer resistance and refractory cancer (Buchser et al., 2012). In mammals, WD-repeat protein interacting with phosphoinositides (WIPI) have been identified as autophagy factors. WIPI members (including WIPI1, WIPI2, WIPI3, and WIPI4) were found to be ubiquitously expressed in normal human tissues but aberrantly expressed in diverse human tumors (Proikas-Cezanne et al., 2004). Recent studies of the WIPI family focused on WIPI1, WIPI2, and WIPI4, while research on WIPI3 mainly concentrated on the function of autophagy and lipid binding (Muller and Proikas-Cezanne, 2015; Bakula et al., 2017; Suleiman et al., 2018; Ji et al., 2020). However, the role of WIPI3 in cancer is completely unknown.

In our work, we integrated the data of cancer gene expression from public database to perform multiple bioinformatics analyses, involving the comparison of WIPI3 expression between HCC and normal liver, detected the influences of WIPI3 mutation on HCC prognosis, and performed functional enrichment of WIPI3-related genes, revealing a new target for diagnosis and treatment of HCC.

## Materials and Methods

### Cell Line and Culture Conditions

Human HCC cell line Huh-7 and normal liver cell line HL-7702 were conserved in our laboratory. Cells were cultured in Dulbecco’s modified eagle medium (DMEM) (Gibco, Carlsbad, CA, United States). The cell media contained 10% fetal bovine serum (FBS, HyClone, Invitrogen), 100 U/ml penicillin, and 100 mg/ml. Cells were maintained in a humidified incubator at 37°C with 5% CO_2_.

### RNA Extraction and qRT-PCR

Cells were collected, and total RNA was prepared using Trizol Reagent (Invitrogen) according to the manufacturer’s instructions. First-strand complementary DNA was synthesized from equal amounts of total RNA (4 μg) using Hifair^®^ III 1st Strand cDNA Synthesis SuperMix for qPCR (11141ES10, TAKARA) and analyzed by Lightcycler 480 SYBR green I master supermix (CW0659S, CWBIO) incorporation in PCRs involving specific primers (Table 1) and performed in a real-time qPCR system (Rotor-Gene3000). The expression level was also calculated using the 2^–Δ^ ^Δ^ ^Ct^ method.

**TABLE 1:** qRT-PCR primer sequences.

Primer name	Sequence 5′→3′
WIPI3	F: GGAGGAGTTGGCCATGTTGAA
	R: CCACAATTCTATCTCGCCGC
GAPDH	F: GGAGCGAGATCCCTCCAAAAT
	R: GGCTGTTGTCATACTTCTCATGG

### mRNA Expression Analysis

We collected the mRNA expression of WIPI3 in HCC through the platform of Oncomine^1^, as well as the DNA copy number variations (CNVs) (Rhodes et al., 2004). Oncomine is currently the largest oncogene chip database in the world, containing 715 datasets and data from 86,733 samples, constituting a conveniently integrated data-mining platform. This analysis was based on a series of researches about HCC, including Chen Liver, Roessler Liver, Roessler Liver 2, The Cancer Genome Atlas (TCGA) Liver, Guichard Liver, and Guichard Liver 2 (Chen et al., 2002; Roessler et al., 2010, 2015; Guichard et al., 2012). The expression of WIPI3 was compared between HCC tissues and normal tissues, and the difference is considered significant when *P* < 0.0001.

Meanwhile, two microarray datasets GSE89377 (Shen et al., 2018) and GSE87630 (Woo et al., 2017), which include 35 and 64 HCC patients, respectively, were analyzed by GEO2R software^2^ for external validation (Davis and Meltzer, 2007). The normalized expression matrix of microarray data could be directly downloaded from the dataset. The probes were annotated by using the corresponding annotation files from the dataset as well.

### UALCAN Analysis

UALCAN is a comprehensive web portal^3^ that allows deep analyses of TCGA gene expression data by using TCGA level 3 RNA-seq and clinical data. One of the UALCAN functions is that it allows researchers to analyze the relative expression of certain genes in tumors and normal samples and in various tumor subgroups such as gender, cancer stages, tumor grade, and other clinicopathological features. We also could use UALCAN database to analyze the prognostic of WIPI3 in HCC (Chandrashekar et al., 2017).

### cBioPortal Analysis

cBio Cancer Genomics Portal^4^ is an open-access resource that can be used to interactively explore multidimensional cancer genomics datasets (Cerami et al., 2012; Gao et al., 2013). We used cBioPortal to analyze WIPI3 mutation in the TCGA liver hepatocellular carcinoma (LIHC) sample (study ID, LIHC_TCGA_Firehouse Legacy), and the “OncoPrint” tab displayed a general view of genetic alterations within each sample in WIPI3.

### LinkedOmics Analysis

The LinkedOmics database is used to analyze 32 TCGA cancer-associated multidimensional datasets, visited at http://www.linkedomics.org/login.php (Vasaikar et al., 2018). The differentially expressed genes related to WIPI3 were screened from the TCGA LIHC cohort (*n* = 371) through the LinkFinder module in the database, and the correlation of results was tested by the Pearson correlation coefficient. The pathway and network analyses of differentially expressed genes were performed by the LinkInterpreter module, the results of which were signed and ranked through the Kyoto Encyclopedia of Genes and Genomes (KEGG) pathways (Kanehisa and Goto, 2000) and gene set enrichment analysis (GSEA) containing cellular component (CC), biological process (BP), and molecular function (MF) (Harris et al., 2006). After 500 simulations, *P* < 0.05 was set as the rank standard.

### UCSC Xena

The heat maps of WIPI3 and five hub genes in the same patient cohort were constructed through data mining in TCGA LIHC by using the UCSC Xena browser^5^ (Zweig et al., 2008; Casper et al., 2018; Goldman et al., 2020).

### Gene Expression Profiling Interactive Analysis

Gene Expression Profiling Interactive Analysis (GEPIA) is a public online database^6^ including a large number of tumors and normal samples for mining cancer data based on TCGA and the GTEx projects (Tang et al., 2017). We used the GEPIA database to analyze the prognostic of WIPI3 in HCC. Based on the specific data group expressed in the TCGA dataset, we analyzed the correlation between the expression of both WIPI3 and the target genes by Spearman’s rank correlation coefficient. The specific parameters were set as follows: the *X*-axis represented WIPI3, the *Y*-axis represented other target genes, and their expression levels in tumors and normal tissues served as variables.

### Establishment of Interactive Network and Modules

The interaction between proteins could be sought through the STRING database, accessed through http://string-db.org, and we screened out coexpressed genes with interaction scores greater than 0.4 to establish a protein–protein interactive (PPI) network (Szklarczyk et al., 2019). The PPI network was then visualized by Cytoscape software version 3.4.0 (Smoot et al., 2011), in which we could find the highly connected protein-interactive regions, and cluster them into modules through MCODE plug-in version 1.4.2 in Cytoscape to prepare for the next analysis.

### Functional and KEGG Pathway Enrichment Analysis

The top genes in modules were annotated via the Database for Annotation, Visualization, and Integrated Discovery (DAVID) version 6.8, an online bioinformatics resource for investigators to comprehend the biological meaning behind the given list by the function of annotation, classification, and more (Dennis et al., 2003; Huang et al., 2009a, b; Yang et al., 2019). KEGG pathway enrichment analysis of five hub genes was also performed by the DAVID software. The results were evaluated significantly at *P* < 0.05 statistical level verified by Fisher’s exact test.

### Statistical Analysis

All statistical analyses were conducted using Prism7 (GraphPad Software). Differences were calculated using Student’s unpaired *t*-test. All bar graphs are presented as SE, and *p* values less than 0.05 were considered significant.

## Results

### Expression of WIPI3 in Human HCC

**FIGURE 1:**
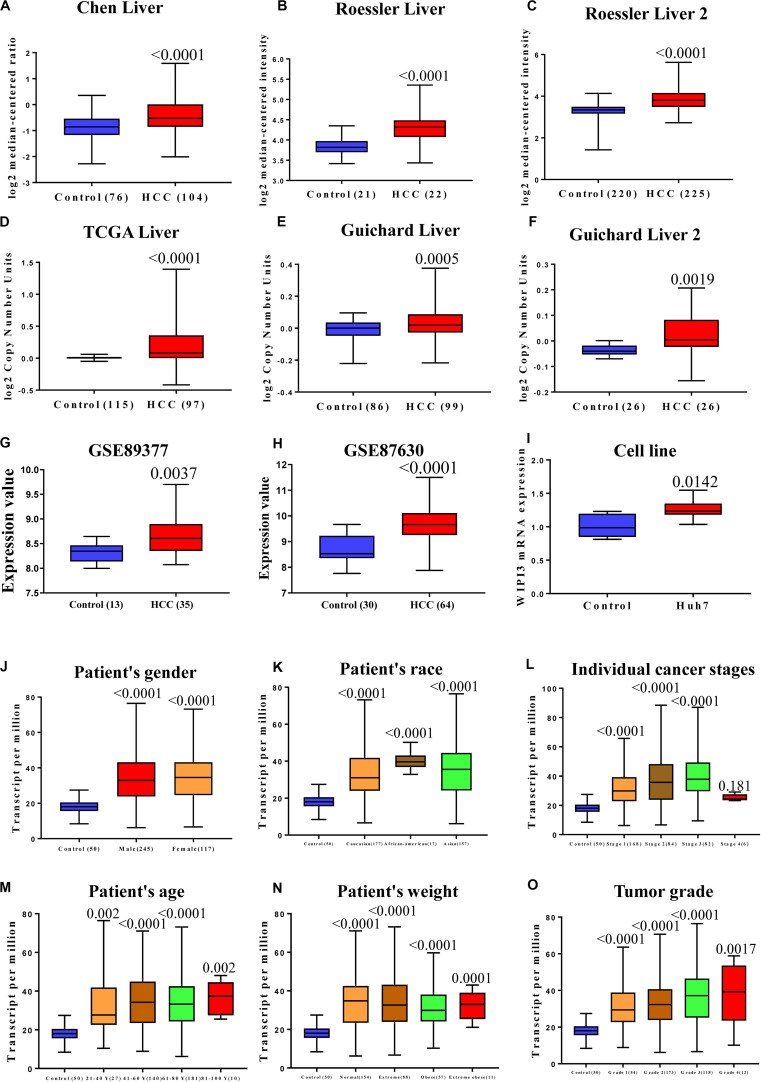
The expression of WD repeat domain phosphoinositide-interacting protein 3 (WIPI3) in human hepatocellular carcinoma (HCC). Box plot showing associated *p* values based on Oncomine analysis of WIPI3 mRNA levels in the Chen Liver, Roessler Liver, and Roessler Liver 2 **(A–C)**. Box plot showing WIPI3 copy number in The Cancer Genome Atlas (TCGA) Liver, Guichard Liver, and Guichard Liver 2 **(D–F)**. WIPI3 expression was analyzed in GEO datasets: GSE89377 and GSE87630 **(G,H)**. mRNA expression of WIPI3 in human HCC cell line Huh7 and human normal liver HL-7702 **(I)**. WIPI3 transcription in subgroups of patients with hepatocellular carcinoma, stratified based on gender, age, and other criteria (UALCAN). The expression of WIPI3 **(J)** in normal individuals of either gender or male or female liver hepatocellular carcinoma (LIHC) patients; **(K)** in normal individuals of any ethnicity or in LIHC patients of Caucasian, African-American, or Asian ethnicity; **(L)** in normal individuals or in LIHC patients in stages 1, 2, 3, or 4; **(M)** in normal individuals of any age or in LIHC patients aged 21–40, 41–60, 61–80, or 81–100 years; **(N)** in normal individual of any weight or in LIHC patients with normal weight, extreme weight, obese, or extreme obese; and **(O)** in normal individuals or LIHC patients with grade 1, 2, 3, or 4 tumors.

We analyzed the transcription levels of WIPI3 in HCC tumors from a series of studies linked to the Oncomine database and found that the mRNA expression of WIPI3 in HCC tissues was obviously higher compared to normal tissues (*P* < 0.01), as well as CNVs. Figures 1A–F show both CNVs and mRNA expression of WIPI3 among the top 25%. Although the differences were not more than twofold, GEO datasets GSE89377 and GSE87630 as test datasets confirmed it (Figures 1G,H). We found a difference in the expression of WIPI3 between HCC and normal liver tissues by analyzing data from the Oncomine and GEO database. Comparing HCC cell line Huh-7 with normal liver cell line HL-7702, we observed an identically significant difference in WIPI3 expression (Figure 1I). To further prove the specificity of WIPI3 in HCC, we integrated various clinical factors of liver hepatocellular carcinoma (LIHC) samples in the TCGA database, for example, gender, race, age, weight, TP53 mutation status, cancer stages, and tumor grade, to compare the transcription levels of WIPI3 in each group. The results showed that WIPI3 in HCC patients still maintained higher transcription levels compared with normal subjects (Figures 1J–O). Thus, the expression level of WIPI3 could be identified as a potential indicator on HCC diagnosis.

### High Expression of WIPI3 and Its Mutations Predict Poor Prognosis of HCC

**FIGURE 2:**
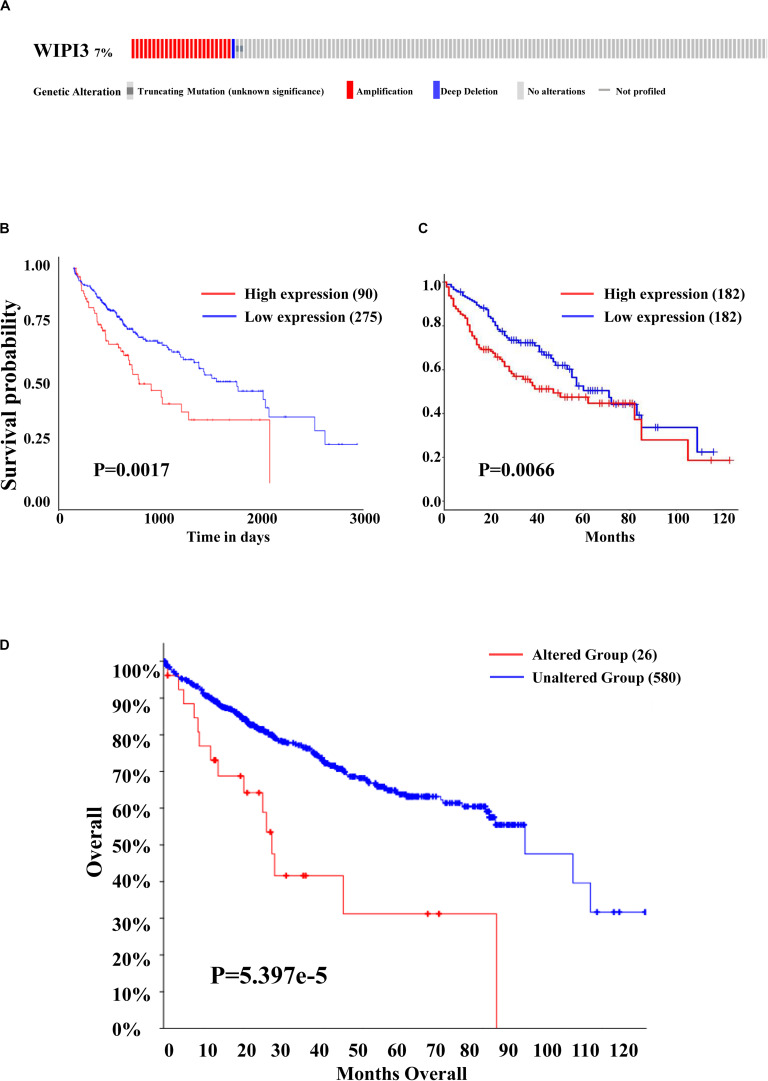
Prognosis and gene alteration of WIPI3 in HCC. **(A)** The OncoPrint schematic showed that gene alteration of WIPI3 occurred in 27 (7.16%) of all 377 sequenced cases, including truncating mutation in 2 cases (0.53%), amplification in 24 cases (6.37%), and deep deletion in 1 case (0.27); Kaplan–Meier (KM) survival curves for overall survival in normal and overall LIHC patients from the UALCAN database **(B)**, the Gene Expression Profiling Interactive Analysis (GEPIA) database **(C)**, and between gene alteration of WIPI3 **(D)**.

We then investigated the prognostic value of WIPI3 in HCC by UALCAN and GEPIA database. As shown by the Kaplan–Meier (KM) curve, there was a close relationship between the expression of WIPI3 and the overall survival of HCC patients that the high expression of WIPI3 caused significantly poor overall survival (*P* < 0.01), especially in 3–4 months (Figures 2B,C). Then, we evaluated the frequency of WIPI3 mutations in 377 sequencing data of LIHC patients in the TCGA database through cBioPortal. There were 27 cases of WIPI3 mutations (7.16%), mainly accounted by amplification in 24 cases (6.37%), and the remaining were truncating mutation in 2 cases (0.53%) and deep deletion in 1 case (0.27%) (Figure 2A). Next, the KM curves analysis of the prognostic value of WIPI3 mutation in HCC indicated that the altered group displayed worse overall survival than the unaltered group (*P* < 0.0001) (Figure 2D). As a result, the mutations of WIPI3 could be one of the predisposing factors for high mortality of HCC.

### Coexpression Genes Correlated With WIPI3 in HCC

**FIGURE 3:**
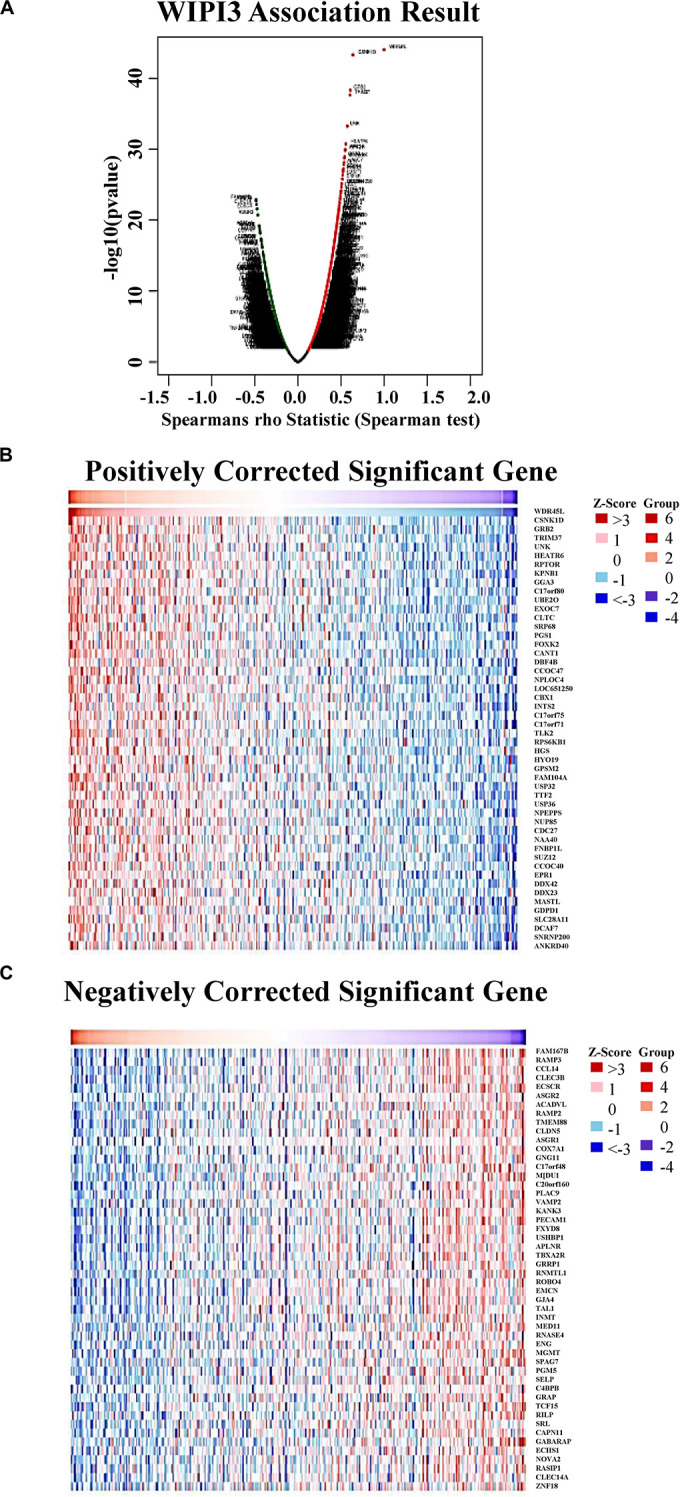
Genes differentially expressed in correlation with WIPI3 in hepatocellular carcinoma (LinkedOmics). **(A)** Spearman test was used to analyze correlations between WIPI3 and genes differentially expressed in LIHC. **(B,C)** Heat maps show the top 50 significant genes positively and negatively correlated with WIPI3 in LIHC. Red indicates positively correlated genes, and green indicates negatively correlated genes.

In the TCGA database, we analyzed the coexpressed genes of WIPI3 in 371 LIHC cases through LinkedOmics (Supplementary Table S1). As shown in Figure 3A, there were 4,416 genes, represented by dark red dots, having an obviously positive connection with WIPI3. Conversely, there were 2,177 genes, represented by dark green dots, having a notably negative correlation with WIPI3 [false discovery rate (FDR) < 0.01]. Fifty significant gene sets were shown by the heat map whether they were positively or negatively correlated with WIPI3 (Figures 3B,C). This result suggested that WIPI3 had a widespread influence on the transcriptome.

### Analysis of Gene Ontology and KEGG Pathways of WIPI3-Related Coexpressed Genes in HCC

**FIGURE 4:**
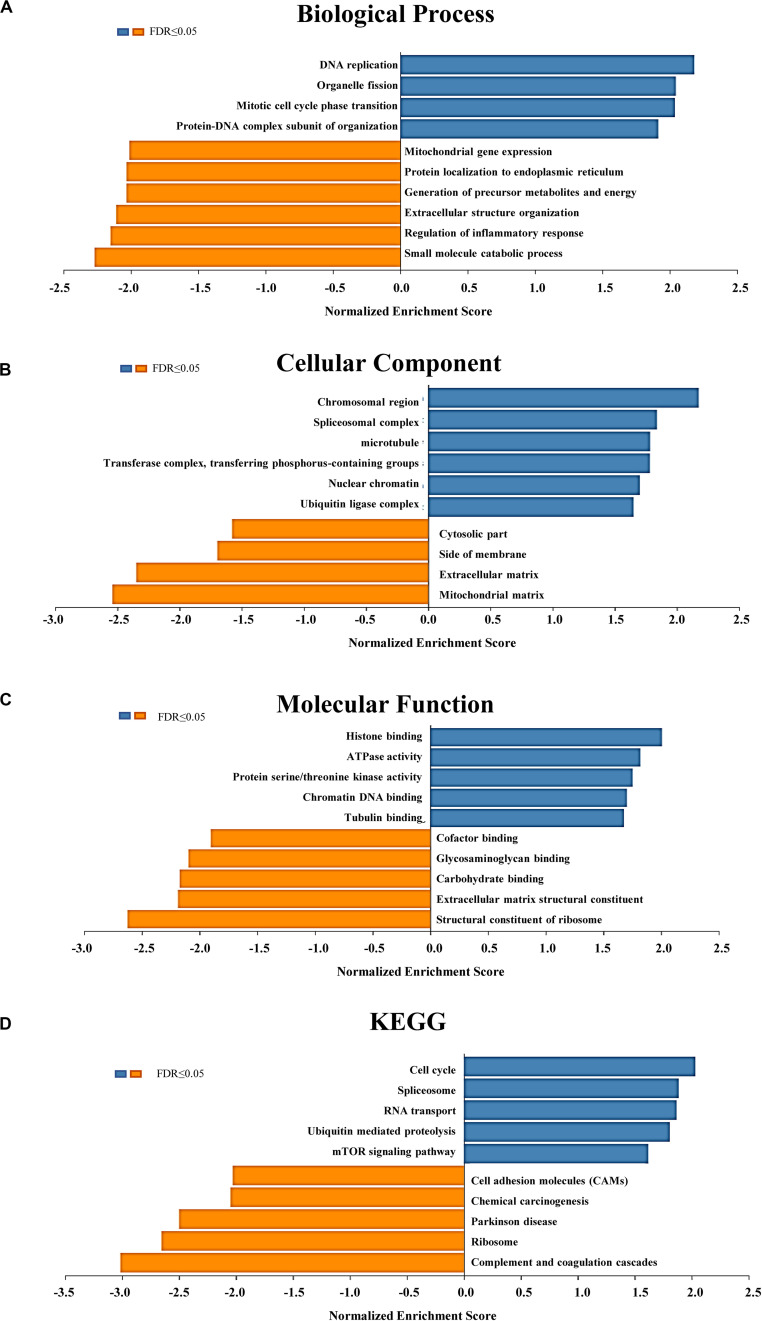
Significantly enriched Gene Ontology (GO) annotations and Kyoto Encyclopedia of Genes and Genomes (KEGG) pathways of WIPI3 in hepatocellular carcinoma. The significantly enriched GO annotations and KEGG pathways of WIPI3 coexpression genes in LIHC were analyzed using gene set enrichment analysis (GSEA). **(A)** Biological processes. **(B)** Cellular components. **(C)** Molecular functions. **(D)** KEGG pathway analysis. The *x*-axis represents the normalized enrichment score, and the *y*-axis represents the term of GO.

The outcomes of Gene Ontology (GO) analysis carried out by GSEA in LinkedOmics indicated that differentially expressed genes correlated with WIPI3 were mainly located in the chromosomal region, spliceosomal complex, microtubule, transferase complex, nuclear chromatin, and ubiquitin ligase complex, where they primarily participated in DNA replication, organelle fission, mitotic cell cycle phase transition, and protein–DNA complex subunit organization. They acted as structural constituents in histone binding, ATPase activity, protein serine/threonine kinase activity, chromatin DNA binding, and tubulin binding (Figures 4A–C). The functions of these differentially expressed genes were principally enriched in the cell cycle, spliceosome, RNA transport, ubiquitin-mediated proteolysis, and mTOR signaling pathways through the KEGG pathway analysis (Figure 4D).

### Construction of Coexpression Gene PPI Network

**FIGURE 5:**
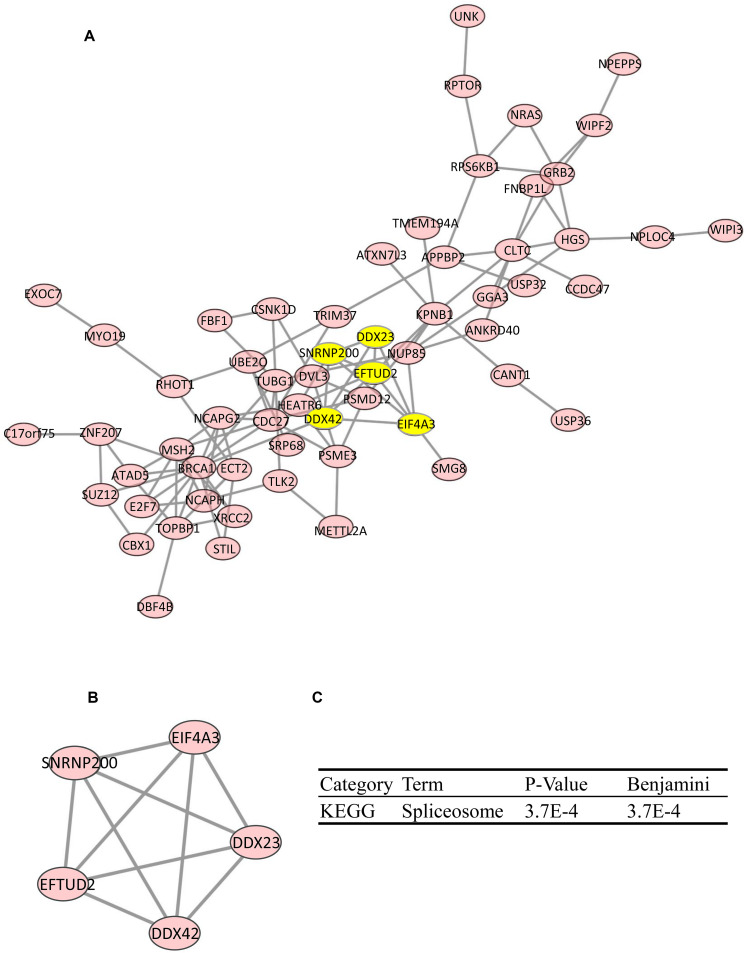
Protein–protein interaction (PPI) network of coexpression gene (top 100). PPI network **(A)** and MCODE analysis indicating the five hub genes DDX23, EFTUD2, EIF4A3, SNRNP200, and DDX42 were identified **(B)**. **(C)** KEGG analysis of five hub genes by Database for Annotation, Visualization, and Integrated Discovery (DAVID).

By using the STRING database, the top 100 significantly coexpressed genes were built into a protein–protein network, and Cytoscape (MCODE plug-in) was used to establish the most important module (Figure 5A). Based on the degree score, the module with the highest score consisting of DDX23, EFTUD2, eukaryotic initiation factor 4A-III (EIF4A3), SNRNP200, and DDX42 was identified as potential hub genes (Figure 5B), highlighted in yellow (Figure 5A). KEGG pathway analysis of hub genes was further performed by the DAVID database. As the result indicated in Figure 5C, the spliceosome pathway was notably affected by hub genes, and the aberrant splicing might lead to gene mutations, which, in turn, had a great influence on protein translation during cell division.

### Analysis of Five Hub Genes

**FIGURE 6:**
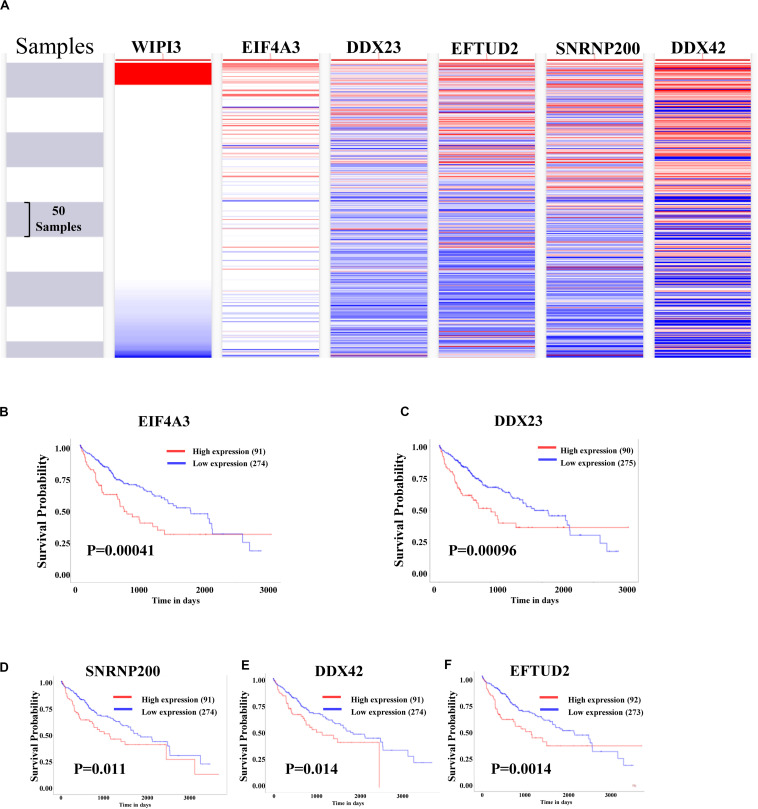
Analysis of WIPI3 and five hub genes in hepatocellular carcinoma. **(A)** The hierarchical clustering of hub genes was constructed using UCSC online database. **(B–F)** Over survival analyses of hub genes in HCC. The results based on the KM plotter database indicate that all hub genes are associated with poor prognosis in HCC.

**FIGURE 7:**
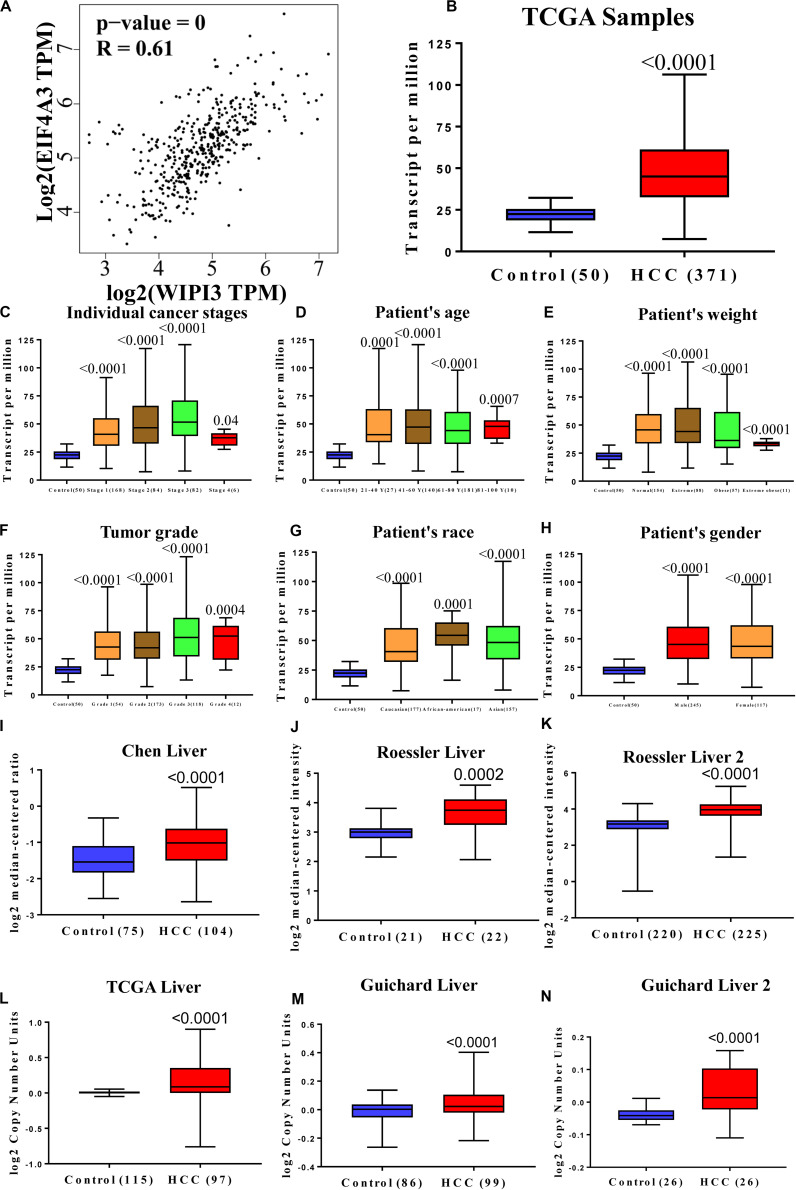
The expression of EIF4A3 is upregulated in hepatocellular carcinoma and highly correlated with WIPI3. **(A)** Correlation between WIPI3 and EIF4A3 mRNA expression in HCC determined using GEPIA. The expression of EIF4A3 **(B)** in normal and LIHC samples; **(C)** in normal individuals or in LIHC patients in stages 1, 2, 3, or 4; **(D)** in normal individuals of any age or in LIHC patients aged 21–40, 41–60, 61–80, or 81–100 years; **(E)** in normal individual of any weight or in LIHC patients with normal weight, extreme weight, obese, or extreme obese; **(F)** in normal individuals or LIHC patients with grade 1, 2, 3, or 4 tumors; **(G)** in normal individuals of any ethnicity or in LIHC patients of Caucasian, African-American, or Asian ethnicity; **(H)** in normal individuals of either gender or male or female LIHC patients. Box plot showing associated *p* values based on Oncomine analysis of EIF4A3 mRNA levels in the Chen Liver, Roessler Liver, and Roessler Liver 2 **(I–K)**. Box plot showing EIF4A3 copy number in The Cancer Genome Atlas (TCGA) Liver, Guichard Liver, and Guichard Liver 2 **(L–N)**.

Using the UCSC Cancer Genomics Browser to hierarchically cluster the five hub genes with WIPI3, it was found that the expression pattern between WIPI3 and EIF4A3 gene was consistent (Figure 6A). Besides, the overall survival of hub genes in HCC was analyzed by the KM curve, which indicated that all five hub genes displayed severe decline in the overall survival rate in higher expression groups (Figures 6B–F). Moreover, there were existing high correlation coefficients between WIPI3 and EIF4A3 through GEPIA analysis (Spearman’s correlation = 0.61) (Figure 7A). Therefore, EIF4A3 might be the most attractive target in spliceosome and cell cycle among hub genes.

### High EIF4A3 Expression Predicts Unfavorable Prognosis in HCC Patients

Subsequently, the expression of EIF4A3 at cancer stages and tumor grades in the TCGA database showed that EIF4A3 was overexpressed in HCC patients compared with normal people (Figures 7B–H). We screened a series of datasets from the Oncomine database, such as Roessler Liver, Chen Liver, and Roessler Liver 2, as well as CNVs in TCGA Liver, Guichard Liver, and Guichard Liver 2 (Figures 7I–N), to identify the expression of EIF4A3 in the subtypes of HCC. The result showed that the upregulation of EIF4A3 expression nearly existed in all subtypes of HCC.

## Discussion

WIPI3, a member of the WIPI protein family, is involved in cell autophagy pathway and lipid binding (Bakula et al., 2017), but its role in cancer remains completely unclear. Here, we screened out available datasets associated with HCC from public databases to confirm the function of WIPI3 on the oncoming, progression, and prognosis of HCC and verified it by HCC cell line and human normal liver cell line *in vitro*. In this study, the expression of WIPI3 remained at a high level with the emergence and development of HCC, leading to poor prognosis, which suggested that WIPI3 could be identified as a potential indicator of HCC diagnosis. We also displayed the mutation of WIPI3 in HCC by cBioPortal. Although the frequency of WIPI3 was altered very low, the WIPI3 altered group displayed a poor prognosis in HCC. Finally, the coexpression genes related to WIPI3 in HCC were analyzed through the LinkedOmics database. GSEA function indicated that coexpression genes participated in cell cycle and spliceosome in GO and KEGG pathway analysis. EIF4A3, filtered from the coexpression genes, could be a partner of WIPI3 in HCC to regulate the development of HCC.

The clinical diagnosis of HCC at an early stage is a severe challenge. At present, the most common indicator at early screening of HCC is alpha-fetoprotein (AFP), but not all HCC patients are AFP positive (Luo et al., 2018). Hence, it is very necessary to find a more effective marker to improve the accuracy of the clinical diagnosis of HCC. The analysis of the transcription levels of WIPI3 in HCC clinical cases from the TCGA database, GEO database, and human HCC cell line showed that mRNA transcription levels and CNVs of the target gene in HCC were significantly higher than normal. The fold change is similar in various HCC studies, although within two instances, WIPI3 ranks in the top 25% of all upregulated genes in HCC based on CNVs. The overexpression of WIPI3 occurs in many HCC cases, which deserves clinical verification as a potential marker in HCC diagnosis and prognosis.

Copy number variations may have important implications on genome, destroying genes and altering genetic content, causing phenotypic differences (Urrutia et al., 2018). In our study, there are an increasing copy number of WIPI3 in the genetic data of HCC, and the main type of WIPI3 mutations is amplification that is tightly related to shorter survival. Hence, we suppose that changes in WIPI3 expression and WIPI3 dysfunction in HCC may be caused by alterations in chromosome structure. The neighboring gene networks close to WIPI3 usually display varying degrees of amplification in HCC, and the related functional networks involve cell cycle, spliceosome, RNA transport, and ubiquitin-mediated proteolysis signaling. Therefore, the WIPI3-altered network is in the key nodes of posttranscriptional regulation, mainly reflected in the process of RNA splicing and protein translation. These results give us more unexpected understanding of the physiological function of WIPI3.

The importance of WIPI3 in the pathogenesis of HCC is proved from the perspective of genomic stability and gene expression. Our study shows that the high expression of WIPI3 in HCC has a profound influence on genome stability as well as on multiple steps of gene expression (spliceosome, RNA transport, and proteolysis) and cell cycle. We used a series of bioinformatics tools for neighbor gene analysis of tumor data from public databases. As a result, WIPI3 is specifically related to several spliceosome complex components (such as DDX23, EFTUD2, EIF4A3, SNRNP200, and DDX42).

EIF4A3, due to its helicase activity in RNA (Linder and Jankowsky, 2011), participates in numerous biological processes associated with RNA, such as RNA transcription, RNA translation, and mRNA decay (Rocak and Linder, 2004; Gehring et al., 2009). EIF4A3 is a key component of the exon junction complex (EJC), and its assembly is associated with splicing (Sauliere et al., 2012). In cancer cells, EIF4A3 is associated with posttranscriptional modification, cell cycle progression, and acceleration of cell growth (Han et al., 2016; Mazloomian et al., 2019; Zheng et al., 2020). Especially in HCC, EIF4A3 is regarded as one of the important phosphoproteins in HCC metastasis (Tian et al., 2015).

The cooperative regulation of EIF4A3 and WIPI3 in cancer cells may indicate a special spliceosome mechanism that can synchronize rapid cell proliferation based on the maintenance of genomic stability. Cotargeting these two collaborative proteins might be an effective treatment for HCC. In conclusion, we have confirmed the upregulation of WIPI3 and its partner EIF4A3 in HCC and verified their importance as prognostic factors. Our research suggests that WIPI3 may be a promising molecular target for the diagnosis and treatment of HCC.

## Conclusion

This study systematically integrated public sequencing data to guide the research of WIPI3 in HCC. It was found that WIPI3 was highly expressed in HCC, which predicted a poor prognosis. The coexpressed gene of WIPI3 in HCC is closely associated with cell cycle and spliceosome, which causes WIPI3 mutations that are leading to lower mortality in HCC patients. Our work shows that EIF4A3 is an important partner of WIPI3 in HCC and suggests that WIPI3 may be an important potential biomarker for HCC.

## Data Availability Statement

The datasets presented in this study can be found in online repositories. The names of the repository/repositories and accession number(s) can be found in the article/Supplementary Material.

## Author Contributions

QK and TW designed and conceived the research. TL did the bioinformatics analysis and wrote the manuscript. QS collected the datasets. ZD searched the literatures. All authors approved the final manuscript.

## Conflict of Interest

The authors declare that the research was conducted in the absence of any commercial or financial relationships that could be construed as a potential conflict of interest.

## Footnotes

1 www.oncomine.org

2 http://www.ncbi.nlm.nih.gov/geo/

3 http://ualcan.path.uab.edu

4 http://cbioportal.org

5 http://xena.ucsc.edu/

6 http://gepia.cancer-pku.cn/index.html
